# Supplementary material for: Quantification of Circulating Cell Free Mitochondrial DNA in Extracellular Vesicles with PicoGreen™ in Liquid Biopsies: Fast Assessment of Disease/Trauma Severity
Source: Cells. 2021 Apr 6;10(4):819. doi: 10.3390/cells10040819 (PMC8067453; doi:10.3390/cells10040819)
Supplement: Supplementary file 1 [file cells-10-00819-s001.pdf]

Supplementary Figures.

Quantification of Circulating Cell Free Mitochondrial DNA in Extracellular Vesicles with PicoGreen in Liquid Biopsies: Fast Assessment of Disease/Trauma Severity.

Michela Marcatti[1], Jamal Saada[2], Ikenna Okereke[3], Charles E. Wade[4], Stefan H. Bossmann[5], Massoud Motamedi[6], and Bartosz Szczesny[2,6,*]

Departments of [1]Neurology, [2]Anesthesiology, [3]Surgery, and [6]Ophthalmology and Visual Sciences, University of Texas Medical Branch, Galveston, TX, USA; [4]Department of Surgery, University of Texas Health Science Center in Houston, Houston, TX, USA; [5]Department of Cancer Biology, The University of Kansas Medical Center, Kansas City, KS, USA

[*]Corresponding author: baszczes@utmb.edu (B.S.)

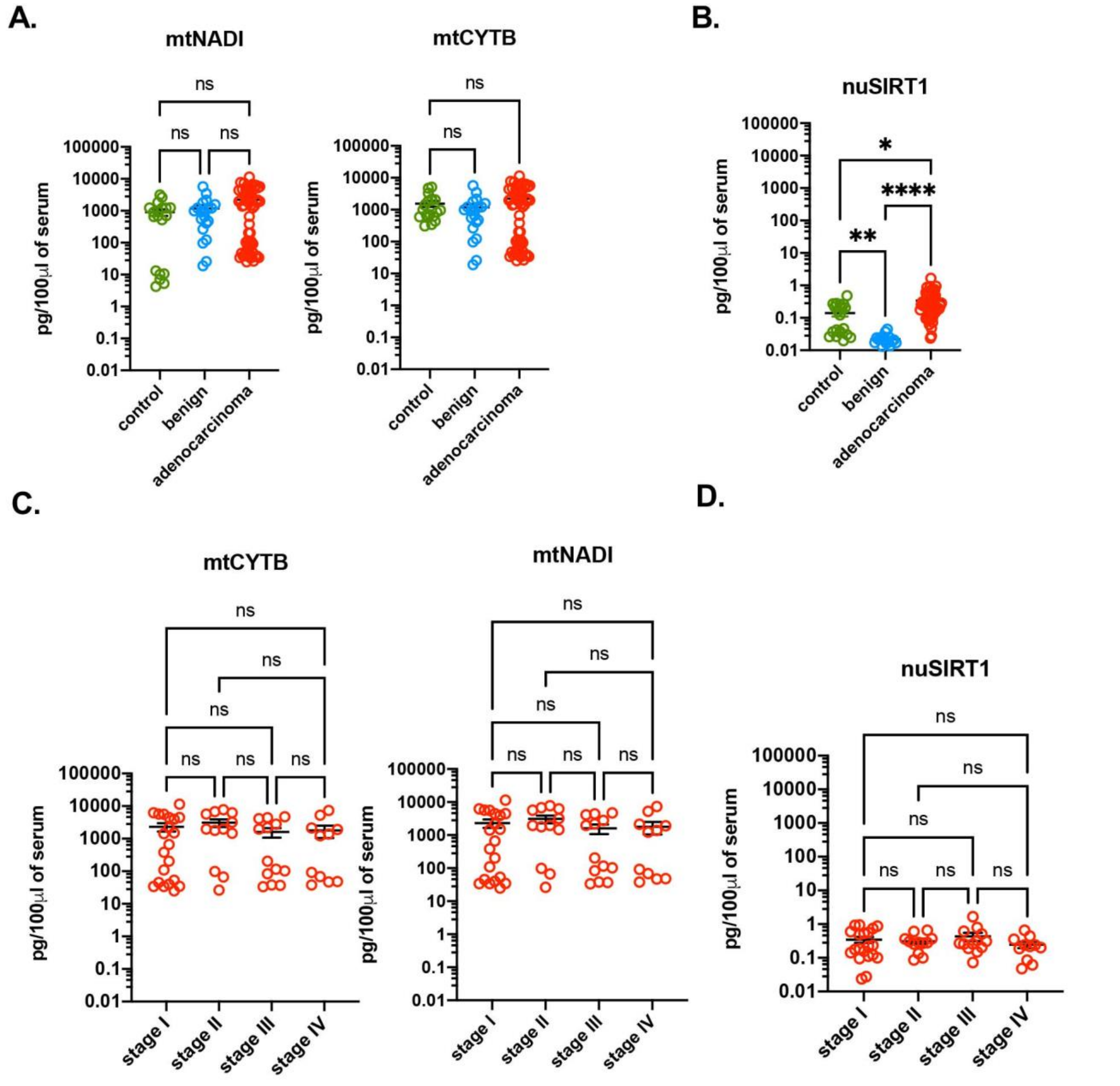

Figure S1. Serum of lung adenocarcinoma subjects have a high amount of ccf-DNA. The amount of mtDNA (A) and nuDNA (B) were quantified with qPCR using mtNADI and mtCYTB, and nuSIRT1 primers, respectively, in serum of healthy volunteers (n=20), subjects with benign tumors (n=20) and with lung adenocarcinoma (n=57). The amount of mtDNA (C) and nuDNA (D) were quantified with qPCR using mtNADI and mtCYTB, and nuSIRT1 primers, respectively, in serum of subject with stage I (n=21), stage II (n=12), stage III (n=13) and stage IV (n=11) of lung adenocarcinoma. The serum was obtained from LCBRN. The amount of DNA is expressed as pg of DNA in 100 µl of serum. * $p<0.05$, ** $p<0.01$, **** $p<0.0001$.

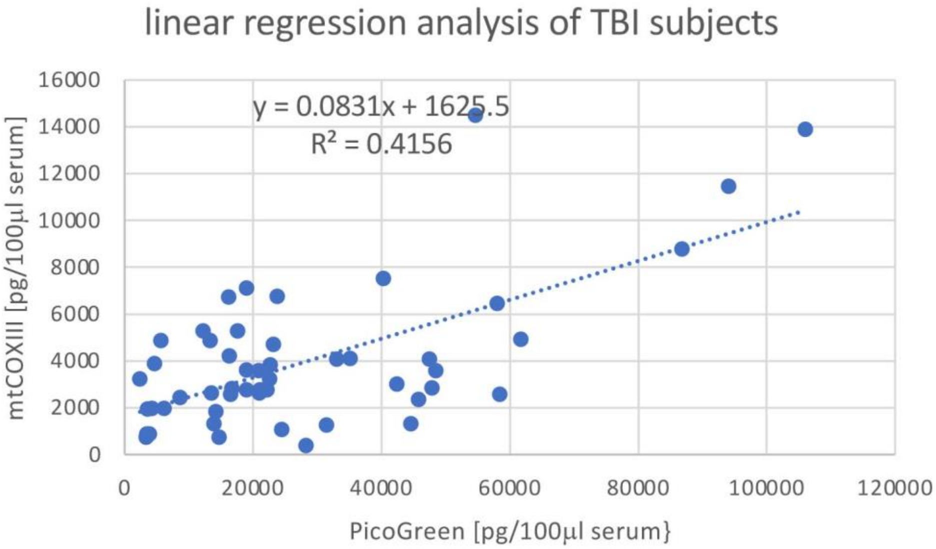

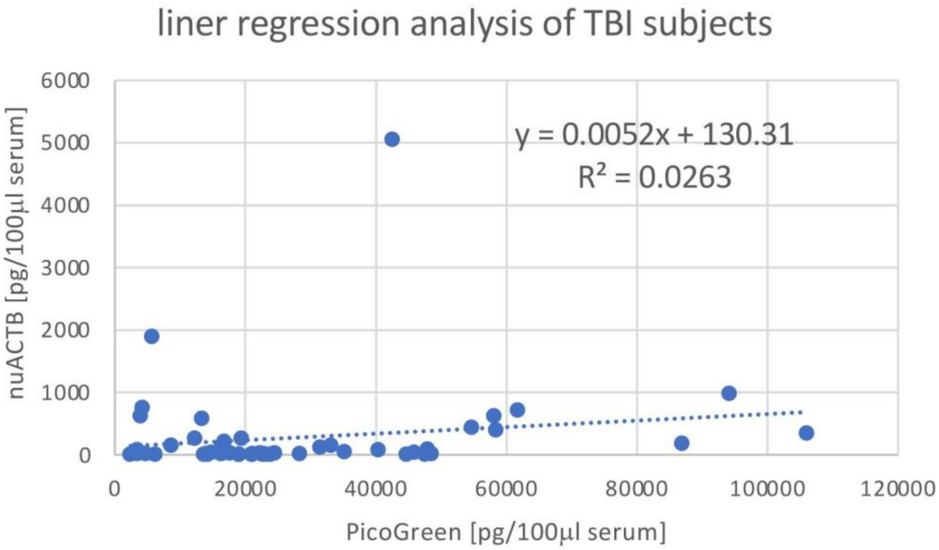

Figure S2. Linear regression analysis of the TBI cohort. We compared amount of ccf-DNA calculated using PicoGreen and with qPCR using mtDNA-specific (mtCOXIII) and nu-DNA-specific (nuACTB) primers. R^2 value was calculated with Excel software.
